# Mass Spectrometry-Based Flavor Monitoring of Peruvian Chocolate Fabrication Process

**DOI:** 10.3390/metabo11020071

**Published:** 2021-01-26

**Authors:** Stephanie Michel, Luka Franco Baraka, Alfredo J. Ibañez, Madina Mansurova

**Affiliations:** 1 Institute of Omics Research and Applied Biotechnology (Instituto de Ciencias Ómicas y Biotecnología Aplicada, ICOBA), Pontificia Universidad Católica del Perú, Lima 15088, Peru; michel.sr@pucp.edu.pe (S.M.); baraka.luka@pucp.pe (L.F.B.); aibanez@pucp.edu.pe (A.J.I.); 2 Post-Graduate Program in Food Technology, Universidad Nacional Agraria la Molina, Lima 15024, Peru

**Keywords:** GC-MS, flavor VOCs, sensory analysis, HS-SPME, monitoring manufacturing process

## Abstract

Flavor is one of the most prominent characteristics of chocolate and is crucial in determining the price the consumer is willing to pay. At present, two types of cocoa beans have been characterized according to their flavor and aroma profile, i.e., (1) the bulk (or ordinary) and (2) the fine flavor cocoa (FFC). The FFC has been distinguished from bulk cocoa for having a great variety of flavors. Aiming to differentiate the FFC bean origin of Peruvian chocolate, an analytical methodology using gas chromatography coupled to mass spectrometry (GC-MS) was developed. This methodology allows us to characterize eleven volatile organic compounds correlated to the aromatic profile of FFC chocolate from this geographical region (based on buttery, fruity, floral, ethereal sweet, and roasted flavors). Monitoring these 11 flavor compounds during the chain of industrial processes in a retrospective way, starting from the final chocolate bar towards pre-roasted cocoa beans, allows us to better understand the cocoa flavor development involved during each stage. Hence, this methodology was useful to distinguish chocolates from different regions, north and south of Peru, and production lines. This research can benefit the chocolate industry as a quality control protocol, from the raw material to the final product.

## 1. Introduction

Chocolate is one of the most popular and recognizable aliments worldwide due to the organoleptic properties of the cocoa beans. Cocoa (*Theobroma cacao L.*) from different geographical origins has a different organoleptic profile and influences the final flavor and quality of chocolate [1,2,3]. The chocolate aroma is primarily due to the volatile organic components (VOCs) from cocoa, composed of a complex mixture of over 500 chemical compounds, mainly pyrazines, esters, amines, amides, acids, and hydrocarbons [4]. Most of these VOCs from cocoa are the foundation for the flavor profile of chocolate products [5,6,7]. Interestingly, besides the cultivation conditions and the genotype (i.e., variety) of the cocoa plant, the fabrication processes can also define the chocolate’s final organoleptic properties [1,4,8], because the relative chemical composition of cocoa’s VOCs is modified during the many different technological processes and metabolic reactions that occur during various stages of chocolate production (e.g., fermentation, drying, roasting, and conching). Therefore, to ensure the chocolate’s final flavor quality, the traceability of cocoa’s geographical origin and the industrial chocolate processing are essential for both the chocolate industry and consumers [9].

The cocoa market distinguishes between “bulk” and “fine-flavor” cocoa (FFC), with bulk cocoa representing 95% of the world cocoa market [10,11]. FFC has opposite features in contrast to that of the bulk cocoa. FFC based chocolates generally have a variable fruity flavor and/or flower/spicy aroma expressions, besides the typical “cocoa” flavor [12,13]. The quality of the FFC and related products, such as chocolates, is done by professional tasters who can also estimate each independent parameter’s intensity (e.g., sweet, floral, sour, etc.) in the sample [14,15,16,17] (i.e., this activity is known as a sensory analysis). The sensory analysis often consists of the deconvolution of the flavor pattern into independent aspects, such as sweet, fresh, ethereal, cocoa, fruity, honey, toasted, caramel, buttery, nut, and floral.

In South America, highly aromatic cocoa beans can be found. Specifically, Peru is a center of diversity of cocoa and several varieties of cocoa have been identified [18,19,20]. Therefore, it is one of the leading exporters of FFC beans in the world [21], and it has the potential to become one of the most prominent countries in the world of high-end chocolate. Hence, identifying these chemical compounds responsible for giving the chocolate its unique flavor and monitoring them during the chocolate-making process is beneficial to increase its final value. 

Consequently, many manufactures are interested in reproducibility of the flavor of their chocolate production, and making it not only recognizable by logo etiquette but also by unique taste. From a scientific point of view, flavor profile is one of the characteristic descriptions of the cocoa species in general [2,4,22,23,24,25,26]. For this reason, a great variety of analytical methods to analyze the organic compounds responsible for the flavor and aroma have been developed [3,6,27].

Mass spectrometry (MS) has been used in many studies for analyzing volatile compounds [28,29,30]. Gas chromatography coupled to mass spectrometry (GC-MS) is particularly suited for analyzing cocoa and chocolate volatiles [26,27,28,31,32,33,34]. In addition, headspace solid-phase microextraction (HS-SPME) has been used to determine the volatile fingerprint of the cocoa bean and chocolate [20,35]. This technique has been reported to be solventless, fast, and highly reproducible, with low limits of detection and increased sensitivity [16,27]. 

This work aims to identify key volatile organic compounds (key-VOCs) of chocolate produced from FCC cultivated beans in northern Peru (i.e., Piura region) and evaluate how key-VOC pattern develops during the elaboration of chocolate bars through an analytical protocol, which is useful to ensure good manufacturing practices. In addition, this research compares GC-MS and sensory analysis results to understand how GC-MS can interpret tasters’ results.

## 2. Results

### 2.1. Identification of Volatile Compounds from Northern Peruvian Chocolate

Ninety-three compounds were putatively identified by HS-SPME-GC-MS analysis. These are shown by organic compounds families and retention time in Table 1. The two most intense mass spectrum (MS) ions fragments (m/z) for each compound and the sensory attribute reported in the literature of some of the chemical compounds are also shown.

### 2.2. Flavor VOCs from Northern Peru

Of the 93 volatile compounds identified (Table 1), the VOCs that presented a significantly higher relative intensity in the chocolate from northern Peru compared to southern Peru were selected. For this purpose, the analysis of variance (ANOVA) was performed. Those MS signals that presented a value of *p* < 10^−6^ were selected because higher *p*-values were not providing enough robustness to the method when randomly permutating the number of chocolate samples for determining the FFC bean origin (i.e., northern or southern Peru).

**Figure 1:**
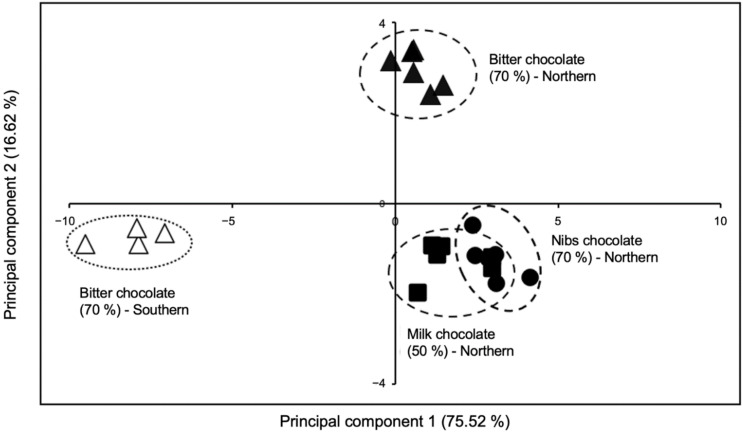
Principal component analysis of chocolates from northern and southern Peru.

In Table 2, the selected key-VOCs are presented according to their retention time and the most intense fragments. These key-VOCs could differentiate chocolate produced with fine flavor cocoa from northern and southern Peru using a multivariate statistical analysis, such as principal component analysis (PCA; Figure 1). The relative intensity pattern of each of the eleven key-VOCs in chocolates from northern and southern Peruvian regions is shown in Supplementary Figure S1. Interestingly, the PCA plot shows four separate clusters. Three clusters in quadrants I and II can refer to northern Peruvian chocolates, while the cluster located in quadrant III refers to chocolate made from cocoa from southern Peru. Furthermore, these key-VOCs can also differentiate three chocolate bars produced with the same FFC beans species from northern Peru due to their different formulation (Figure 1 and Supplementary Figure S1).

By reducing the focus from 93 to 11 VOCs (shown in Table 1 and Table 2, respectively), it is possible to diminish the measurement errors associated with reproducibly monitoring multiple VOCs in food samples [58]. Due to the limited availability of standards, a tandem MS strategy was used for their identification. Thus, the identity of three of them could be validated by their fragmentation pattern measured by a third-party lab using a GC-MS/MS Trace 1300 instrument, Thermo Scientific (MS NIST 2011 Spectral Library). Likewise, since the measured samples are from a known biological origin (i.e., FFC species), peer-reviewed publications of cocoa VOCs were also used to putative identify the metabolites in Table 2. Interestingly, eight of the eleven metabolites in Table 2 have been previously reported to have a flavor description associated with cocoa (Table 1).

**Figure 2:**
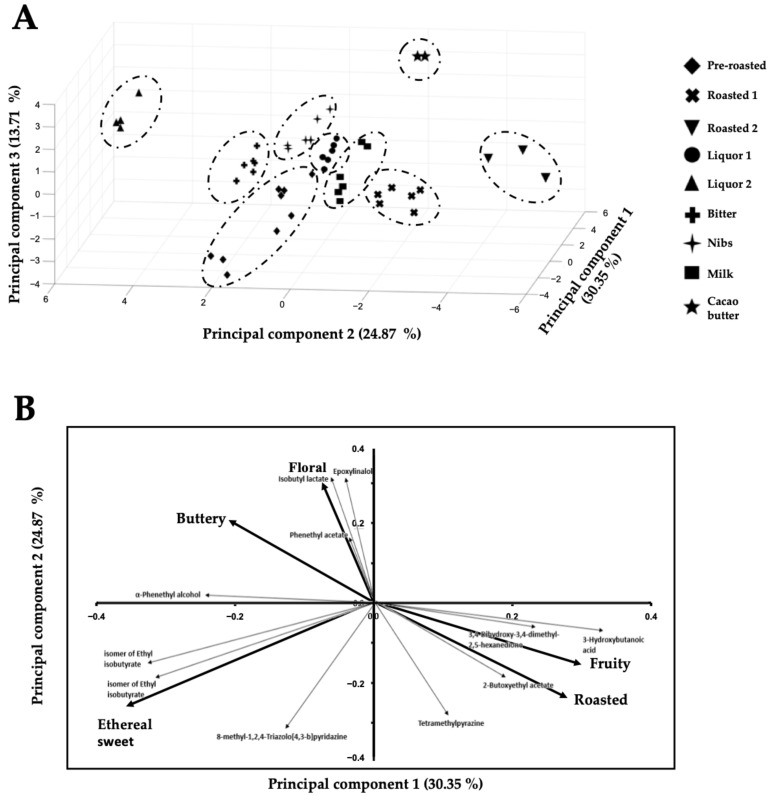
(**A**) 3D—Principal component analysis of key stages of chocolate elaboration and (**B**) Loading plot of the 3D—Principal component analysis of key stages of chocolate elaboration based on (**A**) the eleven key volatile organic compounds.

Furthermore, these eleven key-VOCs can also cluster the chocolate samples based on their manufacturing stages, e.g., pre-roasted beans, roasted beans, conching (liquor prior food additives), and a chocolate bar (Figure 2), the reason being that the relative abundances of these 11 key-VOCs change during the chocolate manufacturing process.

### 2.3. Taster’s Results vs. MS

**Figure 3:**
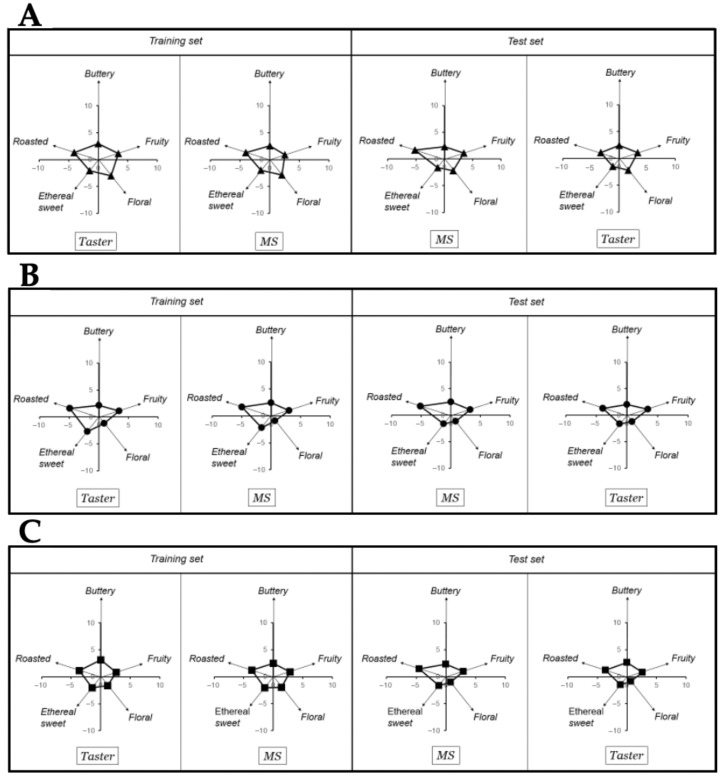
Training and test set of taster results in (**A**) bitter chocolate (70% cocoa), (**B**) nibs chocolate (70% cocoa), and (**C**) milk chocolate (50% cocoa) produced with northern Peru FFC beans.

To ensure that the eleven compounds can be used to monitor the chocolate bars’ flavor, an analogy between the eleven key-VOCs determined by the mass spectrometer and the results from a professional chocolate tasting was made (Figure 3) using a matrix-based polynomial transformation. 

In more detail, for performing this correlation between the eleven key-VOCs and the taster’s results, the key-VOCs were grouped into five classical flavor groups, i.e., roasted, buttery, fruity, floral, and sweet ethereal based on the PCA loading plot (Figure 2). Subsequently, a matrix-based polynomial transformation formula was used to match the normalized MS peak areas with the values provided by human tasters. Thus, a training and test set, each of them consisting in chocolate samples and a panel of human tasters were used. The training set was used to calculate the transformation matrix, while the test set was used to validate the mathematical operation. As both training and test sets chocolate samples measured by the MS could match the panel of professional tasters’ flavor perception used in each set (Figure 3), it is possible to ascertain that the aesthetic flavor perceived from the northern Peruvian chocolates could be inferred from this 11 key-VOCs. 

### 2.4. Flavor Development during Each Key Step of Chocolate Elaboration

**Figure 4:**
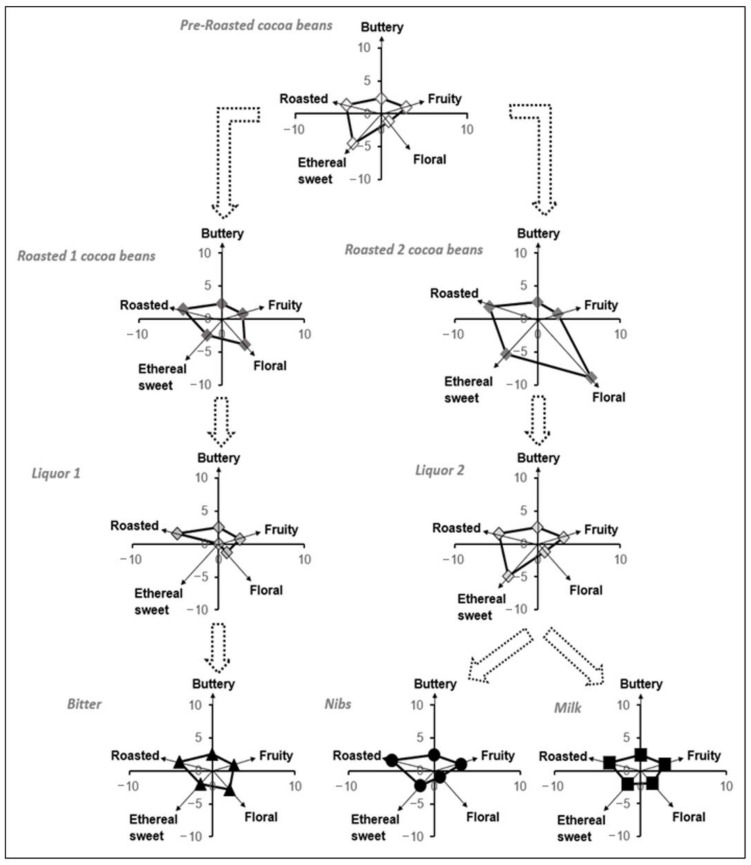
Flavor development during each key step of chocolate elaboration.

With the hypothesis that the eleven key-VOCs could correlate to the chocolate’s flavor, the subsequent step was to study how these VOCs evolved during two of the key chocolate-making stages, such as roasting and conching (Figure 4) using the described GC-MS methodology. Roasting procedures 1 and 2 are done at the same temperature range (between 130 to 160 °C). However, roasting procedure 1 is performed for a shorter time (<30 min), while roasting process 2 takes longer (>30 min). The conching procedures 1 and 2 are similar in terms of temperature and time, the only exception being the type of roasted beans used. For the particular case of the nibs and milk chocolate, the liquor sample was taken before adding other ingredients.

## 3. Discussion

This work’s underlying concept is based on the culinary hypothesis known as food pairing [59]. According to this theory, taste perception is the neuronal response to chemical compounds present in food. Therefore, by characterizing the odors (volatile organic compounds, VOCs, molecules) and the savors (mainly non-volatile molecules) of food, it is possible to predict the taste that a person perceives in a recipe. 

To identify the key volatile organic compounds (VOCs) that can be characteristic for the Peruvian chocolate made from white porcelain FFC beans from northern Peru, ninety-three VOCs were first identified using GC-MS. Table 1 shows the list of all these compounds, where the most predominant are esters, followed by alcohols and phenols. In detail, 6.46% are acids; 18.28% alcohols and phenols, 13.98% aldehydes, and ketones; 19.35% esters; 5.37% furans, furanones, pyranes and pyrones; 4.3% hydrocarbons; 5.37% lactones; 1.07% nitrogen compounds; 13.98% pyrazines and piperazines; 2.15% pyridines; 4.3% pyrroles; 2.16% sulfur compounds and 3.23% terpenes and terpenoids. Esters and alcohols families of organic chemical compounds provide chocolate with a sweet and fruity aroma, very characteristic of chocolates made with FFC beans [37,55]. The aldehydes, ketones, pyrazines, and piperazines are the next families of chemical compounds in a higher proportion that provide a charactersitic flavor. The aroma of roasted cocoa is very characteristic of pyrazines [60,61] and also represents one of the most important flavors of cocoa products [41]. Many of the compounds described in Table 1 have an odor descriptor reported in the literature, but there are still chemical compounds that do not have an associated descriptor. 

Of the ninety-three volatile compounds, eleven were selected as key-VOCs for being highly correlated to the northern Peruvian chocolate. Interestingly, not all of these eleven compounds in Table 2 are unique to the region. Six compounds have been reported as cocoa flavor compounds from West Africa (e.g., tetramethylpyrazine, epoxylinalol, phenethyl acetate), Asia (e.g., tetramethylpyrazine, ethyl isobutyrate), and Latinamerica (e.g., tetramethylpyrazine, α-phenethyl alcohol). Nevertheless, as shown in Figure 1, the relative abundance between these VOCs (pattern) is characteristic of the northern Peruvian region.

The PCA plot (Figure 1) based on these eleven key-VOCs (Table 2) shows four separate clusters. It must be noted that each chocolate type (Bitter, Nibs, and Milk) produced with the same harvest of white porcelain FFC beans from Piura (northern region in Peru) has a slightly different clustering quadrant in the PCA due to the industrial process they go through. It will be demonstrated later that this separation among northern chocolate types is mainly due to the difference in the manufacturing conditions and not a particular food additive. 

Interestingly, key-VOCs of “similar” known flavors (Table 2) cluster together in the loading plot (Figure 2B), while key-VOCs of contrasting flavors cluster in different directions in the loading plot. For example, on the one hand, tetramethylpyrazine has the characteristic aroma of roasted cocoa and is also present in chocolates of Ecuador and West Africa [42]. Tetramethylpyrazine is a key roasted aroma contributor to cocoa, with coffee- and cocoa-like attributes [31,33,35]. On the other hand, phenethyl acetate has been reported to have a sweet floral taste [43] as well as α-phenethyl alcohol. Although α-phenethyl alcohol is a volatile compound in milk, it is also present in the cocoa samples [15,16,39,42,55,62]. For the compounds in Table 2 that did not have a reported flavor, we hypothesize that these compounds will share the same flavor characteristics as other “known flavor” compounds that cluster with them. For example, 8-methyl-1,2,4-triazolo[4,3-b] pyridazine, 3-hydroxybutanoic acid, and 3,4-dihydroxy-3,4-dimethyl-2,5-hexanedione were correlated with a sweet and fruity, since their loading plot position clusters them with the putatively identified metabolites ethyl isobutyrate and 2-butoxy ethyl acetate (Figure 2B). Thus, the present method could only monitor five flavors (i.e., buttery, fruity, floral, ethereal sweet, and roasted flavors) associated with our key-VOCs from all possible (10) flavors named in Table 1.

Once all compounds in Table 2 had a putative flavor; a matrix-based polynomial transformation formula was used to match the normalized peak areas of each flavor group with the values provided by the tasters. First, a tasting test of the chocolates was performed (i.e., training set), where the intensity of the 10 flavors, which included the five flavors (i.e., roasted, buttery, fruity, floral, and sweet ethereal) of interest, were identified by two professional tasters (provided by Theobroma Inversiones SAC). The objective was that the human tasters where as specific as possible on the intensity of each flavors they perceived to better their responses with the MS signals.

The objective of the training set was to mathematically construct the flavor pattern using the MS signals. For this purpose, the flavor pattern given by the Human tasters was key for calculating a transformation matrix (see Section 4.6). Subsequently, a new experiment was run (i.e., test set) to validate our estimation of the transformation matrix (see Section 4.6). A test set consists of a new set of chocolate samples measured with our MS approach to predict the human flavor perception values from a new (different) panel of three tasters from Theobroma Inversiones SAC and a third-party institution. In Figure 3, the results of the flavor pattern comparison between both groups (human tasters and GC-MS signals) demonstrates that the MS-based analysis could match the flavor perception of the professional tasters. Thus, we are confident that we could use our 11 key-VOCs to ascertain the chocolates’ human response.

With the certainty that the eleven key-VOCs could represent the flavor that a person can perceive, a polynomial transformation of the relative amounts of these key-VOCs was used to follow the flavor development upon the different stages of chocolate manufacturing since their relative proportions were unique to each stage (Figure 2A, Figure 4). So, during roasting, it was observed that floral and roasted flavors increase when the beans are roasted at higher temperatures. This relative increase is in accordance with the current scientific knowledge available for chocolate manufacturing. The compounds associated with these flavors are products of the Maillard reaction, which occurs during roasting [4,62,63]. Hence, at higher temperatures, more compounds associated with the Maillard reaction can be seen. While during the conching, time and temperature play a pivotal role in releasing the volatile compounds from the liquor. Hence, there is a small decrease in ethereal sweet flavors since they are incredibly volatile. In Figure 4, it is also possible to observe a slight difference between the liquor (conching stage prior additives) and the final product, in particular for the Nibs and Milk chocolate, since they receive additives before the tempering and packaging steps. 

In Figure 4, it is observed that the floral flavor is the least intense in the pre-roasted beans. Here, it was noticed that this flavor’s perception increased upon roasting the beans for the Nibs/Milk chocolate (Roasted beans 2). In contrast, in roasted beans related to Bitter chocolate (Roasted beans 1), the increase is somewhat less meaningful. This difference may indicate that the roasting conditions affect this particular flavor development in the FFC beans [64,65]. In addition, it was observed that the intensities of buttery and fruity flavor were maintained almost constant during both roasting processes of the cocoa beans.

In the conching stage, where the cocoa liquor is obtained, it was observed that the flavors’ intensities that changed significantly were the ethereal sweet for liquor 1 and floral for liquor 2. We estimate that this decrease is associated with frictional heat and the consequent release of volatiles. The loss of flavors such as sweet, floral, and fruity during the conching process has been reported in some studies [47,64,66].

Finally, it is observed that the ratio between the floral/ethereal sweet flavor, which had decreased in intensity in liquor 2, has increased later in the Nibs and Milk chocolate. The explanation for this result lies in the addition of cocoa butter (food additive). Therefore, it can be pointed out that the cocoa butter helps to compensate for the ratio between the floral/ethereal sweet flavors that had strongly diminished after the conching stage (liquor 2).

## 4. Materials and Methods

### 4.1. Materials

Pre and post roasted beans, liquor, and chocolates from northern Peru origin were obtained from Theobroma Inversiones SAC (Lima, Peru). The chocolate samples of Theobroma Inversiones SAC were bitter chocolate (70% content of cocoa) from 5 chocolate bar lots, nibs chocolate (70% content of cocoa mixed with nibs and cocoa butter) from 3 chocolate bar lots, and milk chocolate (50% content of cocoa mixed with powder milk and cocoa butter) from 5 chocolate bar lots. These chocolates were produced from the same species of harvested cocoa fruits obtained in the northern Peru region in 2018 and 2019. The southern Peruvian chocolates used as a comparison were obtained from local supermarkets (70% content cocoa from the southern Peru region). All chocolate bars were analyzed before their expiration date.

Pre and post roasted beans were stored in sealed aluminum paper containers at ambient temperature (18–20 °C). Liquor and chocolates were stored in sealed plastic containers in a fridge (4 °C). Theobroma Inversiones SAC (Lima, Peru) provided us several chocolate bars from their prize-winning product line: Piura Select (cocoa content 70%, named Bitter); Piura Nibs (70%), and Piura Milk (50%). These materials were also analyzed within the expiration date suggested by the company.

### 4.2. Sample Preparation and Volatile Compounds Extraction

One (1) g of each type of chocolate was grated in a mortar to form a fine powder. Then, the chocolate powder was added to a septum vial (20 mL). Volatile compounds of each sample were extracted using the Headspace Solid-Phase Microextraction technique (HS-SPME). The fiber used for the extraction was 50/30 µm divinylbenzene/carboxen/polydimethylsiloxane (DVB/CAR/PDMS, Stableflex 24 Ga, Manual Holder) of Supelco. The use of this fiber for cocoa organoleptic analysis allows obtaining a good separation of chromatographic peaks [41]. The SPME fiber was conditioned in the GC-MS Agilent 7890B’s injector system for 15 min at 250 °C. The conditioning was done below the suggested conditioning temperature provided by Supelco for this fiber type (i.e., 30 min at 270 °C) because it was found that our conditions extended the working life of our fiber without carry over compromise. After fiber conditioning, the SPMEs fibers were exposed to heated chocolate samples (at 60 °C) for 15 min in a thermostat block. Although individuals consume chocolate bars at room temperature, the temperature of 60 °C used was to maximized VOCs emission without risking degradation. The VOCs were then desorbed in the GC-MS Agilent 7890B’s injector system for 10 min at 250 °C.

### 4.3. HS-SPME-GC-MS Method

Chocolate samples were analyzed by gas chromatography—mass spectrometry (GC-MS), using the equipment Agilent 7890B GC System, Equipped with a VF-23ms column (high polarity column, length: 30 m, diameter: 0.25 mm, film thickness: 0.25 µm). The GC inlet was at 250 °C, while the oven was set at an initial temperature of 40 °C for 5 min, then the temperature was increased to 200 °C with a gradient of 5 °C/min, to finally keep at 200 °C for 10 min [41]. Injection mode was performed manually, exposing the fiber after introducing the SPME needle. The fiber was left exposed for about 10 min and then removed from the inlet.

The SPME fiber selection was made by analyzing chocolate samples from three production lots of bitter chocolate samples (70% Cocoa content) from Theobroma Inversiones SAC. Two samples from different production lots are shown in Supplementary Figure S2. The divinylbenzene/carboxen/polydimethylsiloxane (DVB/CAR/PDMS, black line) and the divinylbenzene/polydimethylsiloxane (DVB/PDMS, red line) fibers showed better performance than the carboxen/polydimethylsiloxane (CAR/PDMS, blue line) fiber due to the higher affinity of the latter to acetic acid. The DVB/CAR/PDMS was finally selected because, after 17 min, it showed better chromatographic peaks than DVB/PDMS fiber.

During SPME optimization, every sample was analyzed in triplicate, and blanks (i.e., no-exposed fiber injections) were run between every sample. Once the method was optimized, six additional chocolate samples were analyzed from production lots of two different years, i.e., 2018 and 2019; to identify key-VOCs that can differentiate northern and southern Peru regions. The number of blanks was reduced to 1 every three sample injections.

After obtaining the chromatograms and spectra of each sample, the signals were integrated using the GC-MS software. In each integration, the NIST 2.0 Mass Spectral Search Program database and the MS NIST 2011 Spectral Library were accessed to identify the compounds. This database allowed access to a list of probable compounds according to the percentage of equivalence between experimental and theoretical mass spectra. For the ninety-three chromatographic peaks, the compound’s name with the highest identification percentage of similarity to the mass spectrum was selected (minimum accepted 60%). Low identification percentage is typical with old quadrupole mass analyzer models since it only works with nominal masses. Therefore, to verify the compounds’ identity, we searched as well in peer-review references, where these putative signals were also identified in chocolate samples.

### 4.4. Sensory Analysis

The sensory analysis of the chocolate bars was carried out by the qualified tasters of the company Theobroma Inversiones SAC (4 individuals) and one from a third-party company. The number of tasters used is in agreement with standard practices for an international chocolate/cocoa degustation or contest, where a minimum of three to more tasters is used. Furthermore, two of the authors took steps to be qualified as tasters. However, they were not yet certified at the time of the trials and did not participate in the trials. Nevertheless, they could ensure that the trials were done according to international standards.

The term “qualified (taster)” refers to a person trained for at least four months to deconvolute the cocoa and cocoa-related products’ flavors. The training is based on developing a flavor memory by trying different flavors present in various cocoa-related products. To become an official taster, the candidate must describe the flavors in cocoa and cocoa related products. The candidate results are subsequently compared to those of a certified taster. If the candidates’ results are within a 10% difference of the certified taster score, they become themselves certified tasters as well.

Tasters (5 individuals, divided into two groups) evaluated 52 samples (i.e., 10 to 11 samples per taster). These samples came from four chocolate lots produced with FFC beans from northern (Bitter 70%, Nibs 70%, and Milk 50%) and southern (Bitter 70%) Peru. Each person was provided with a sample of chocolate (from a different origin) and a randomly doubled sample as a control. The attributes selected by the human tasters for evaluation were sweet, fresh, ethereal sweet, cocoa, fruity, honey, roasted, caramel, buttery, nut, and floral. We used a larger number of attributes to facilitate our ability to correlate the human taster’s perception of the key-VOCs measured by the MS.

The samples (approximately 2 g) were placed on aluminum foil and coded with random numbers. The tasters were given water and water cookies to neutralize the palate.

### 4.5. Selection of Five Groups of Flavors

The selection of the five flavor groups was based on the PCA loading plot (Figure 2). As a result, the compounds that did not have a reported taste were given the flavors of the compounds they clustered with. Afterwards, the perceived intensity for these five flavors were correlated to the normalized MS peak areas by applying a third-degree polynomial transformation to the MS signal matrix (Section 4.6).

### 4.6. Experimental Design and Statistical Analysis

The multivariate analysis clustering (i.e., principal component analysis) and the polynomial matrix transformation were performed on MATLAB vR2019b. In more detail, the statistical analysis was used to correlate certain volatile compounds to previously defined chocolate flavors, thus permitting us to correctly identify, with a significant degree of confidence, particular chocolate flavors. The volatile compounds’ signals were normalized to the total ion current (TIC) value of each spectrum to monitor their variations throughout the industrial process of making different types of chocolates. Eleven (11) of the 93 volatile compounds detected in all samples were extremely good VOCs to differentiate the chocolate bars’ origin. Therefore, the number of possible VOCs related flavors observed in Table 1 was reduced using PCA to the observed ones in Table 2 (i.e., buttery, fruity, floral, ethereal sweet, and roasted flavors), providing an excellent starting point for flavor correlation and determining the prize-winning chocolate bars’ secret flavor pattern.

Subsequently, the semi-quantitative correlation between the normalized MS peak areas by TIC and the average human tasters’ flavors was performed by applying a third-degree polynomial transformation to the MS signal matrix. More specifically:

A_i_ × B_i_ = C_i_ (1)

where A_i_ is a 3 × 3 matrix with rows given by normalized MS signals of the metabolites associated with the flavor “i” (Table 2) and columns given by the type of chocolate (e.g., bitter, nibs, and milk); B_i_ is the unknown 3 × 1 transformation matrix for flavor “i”; and C_i_ (1 × 3 matrix) is the average testing values for flavor “i” identify in a given type of chocolate. The matrix operation to identify B_i_ was performed in Matlab. Since multiple signals could be correlated to a particular flavor (Table 2), the above-described process was manually repeated several times by exchanging the selected MS values to obtain the result closest to the human testers.

Finally, to validate our estimation of the transformation matrix (B_i_) for each flavor i, a new set of chocolate samples was measured with our MS approach. The selected normalized MS signals were introduced to the above equation to predict the human flavor perception values from a new (different) panel of tasters (three individuals).

## 5. Conclusions

Our GC-MS based method identified eleven chocolate volatile organic compounds (VOCs) that could indicate: (a) if the chocolate was produced with white porcelain FFC beans from Piura, northern Peru (i.e., origin), and (b) that could infer the perceive flavor of chocolate bars made with white porcelain FFC beans from Piura. More interestingly, it was possible to monitor the changes in the relative abundance of these 11 VOCs through the different stages of chocolate manufacture. We believe that, in the near future, the implementation of these techniques by food security officials may allow them to trace the origin of the chocolate bar and identify adulterations or bad manufacturing practices among fine flavor chocolate producers in Peru.

## Figures and Tables

**Table 1:** Volatile compounds of dark chocolate from northern Peru (*).

Retention Time (min)	Volatile Organic Compound	Odor Description	Fragments (*m/z*)	Ref. ^a^
	**Acids**			
8.046	Acetic acid	Sour, astringent, vinegar	60, 61	[16,27]
11.772	Propanoic acid	Pungent, rancid, soy	74, 73	[27,36]
12.807	Isobutyric acid	Rancid, butter, cheese	73, 88	[16,37]
14.527	Butanoic acid	Rancid, cheese, sweat	60, 73	[27,36,38]
15.651	Isovaleric acid	Sweat, acid, rancid	60, 87	[16,37,39]
22.080	3-Hydroxybutanoic acid		59, 71	
	**Alcohols and Phenols**			
5.049	1-Butanol	Fusel, dry	56, 55	[40]
6.191	1-Ethoxy-2-propanol		59, 61	
9.608	3-Methyl-2nitrobenzyl alcohol		104, 93	
11.280	2-Heptanol	Citrusy, floral	55, 83	[16,41,42]
12.902	2,4-Dimethyl-3-pentanol		73, 55	
14.206	3-Octenol	Earthy, green, oily ^b^	57, 55	[43]
14.409	4-Cyclohexene-1,2-diol		60, 96	
16.991	1-Octanol	Fatty, waxy	55, 56	[37,44]
18.049	cis-3,3,5-Trimethylcyclohexanol	Minty ^b^	83, 109	[43]
18.155	1-Methoxy-2-butanol		59, 58	
18.317	2,4-Dimethyl-3-pentanol		73, 55	
18.730	6-Methyl-1-octanol		55, 105	
19.935	3-Methoxy-1-butanol		59, 71	
22,386	Guaiacol	Smoked, sweet	109, 124	[16,45]
22.570	Benzyl alcohol	Sweet, flower	79, 77	[14,39,41]
23.369	α-Phenethyl alcohol	Sweet, floral	79, 107	[14,16]
27.075	2,2-Dimethyl-1,3-propanediol		56, 55	
	**Aldehydes and Ketones**			
2.671	3-Methyl-2-heptanone		72, 57	
2.935	Isovaleraldehyde	Malty	58, 57	[45]
3.767	Pentanal	Warm, fruity, nutty	58, 57	[14,40,44]
6.548	Hexanal	Green	56, 57	[14,44,45]
9.793	Heptanal	Fatty, harsh, pungent	57, 70	[14,40]
10.027	2-Heptanone	Fruity, spicy, cinnamon	58, 71	[9,14,40]
15.739	Nonanal	Soapy	57, 56	[14,44,45]
15.874	2-Nonanone	Milk, green, fruity	58, 57	[14,36,39]
17.867	2-Cyclopentene-1,4-dione		96, 68	
20.097	Acetophenone	Flower, almond, sweet	105, 77	[9,37,39]
20.790	2-Undecanone	Fruity, waxy	58, 60	[15]
21.434	3-Methylcyclopentane-1,2-dione	Caramel, maple ^b^	112, 55	[43]
23.267	3,4-Dihydroxy-3,4-dimethyl-2,5-hexanedione		88,89	
	**Esters**			
5.469	Butyl acetate	Fruity, pineapple	56, 57	[9,40]
7.228	Isoamyl acetate	Fruity, sweet, banana	70, 55	[39,40]
15.126	(2E)-2-Butenyl propionate		57, 75	
17.346	Isobutyl lactate	Buttery, caramelly ^b^	57, 75	[43]
18.239	2,3-Butanedioldiacetate	Fruity	87, 72	[46]
19.309	2-Butoxyethyl acetate	Sweet, fruity ^b^	55, 88	[43]
20.176	Ethyl benzoate	Sweet, fruity, cherry	105, 77	[15]
20.591	an isomer of Ethyl isobutyrate	Ethereal sweet, fruity	71, 116	[36]
20.824	an isomer of Ethyl isobutyrate	Ethereal sweet, fruity	71, 116	[36]
21.994	2-Ethoxyethyl-3 -methylbutanoate		85, 57	
22.156	Methyl benzeneacetate	Sweet, floral, honey ^b^	91, 150	[43]
24.247	1-Methylbutyl benzoate	Sweet, fruity ^b^	105, 123	[43]
24.578	Phenethyl acetate	Sweet, floral	91, 104	[45]
25.206	Propanoic acid, 2-methyl-2,2-dimethyl-1-(2-hydroxy-1-methylethyl) propyl ester		71, 83	
25.616	Propanoic acid, 2-methyl-,3-hydroxy-2,4,4-trimethylpentyl ester		71,56	
29.117	Triacetin	Creamy, slightly acidic ^b^	103, 145	[43]
30.001	Ethyl-4-ethoxybenzoate		121, 149	
				
30.689	Piperidin-2-one-5-carboxylic acid,5,6-didehydro-,methyl ester		155, 124	
	**Furans, furanones, pyrans, pyrones**			
9.281	2-Pentylfuran	Musty, green	81, 82	[47]
17.222	Furfuryl alcohol	Faint burning	98, 97	[48]
27.307	3,5-Dihydroxy-6-methyl-2,3-dihydro-4H-pyran-4-one	Roasty, caramel	144, 101	[49]
31.019	2,3-Dihydrobenzofuran	Sweet, herbal, floral	120, 91	[50]
31.294	5-Acetyldihydro-2(3H)-furanone	Wine	85, 57	[51]
	**Hydrocarbons**			
3.515	2,2,4,6,6-Pentamethylheptane		57, 56	
3.939	Toluene	Sweet	91, 92	[45]
6.725	Undecane		57, 71	
11.866	1-Methyl-3-propylbenzene	Off odor	105, 74	[52]
	**Lactones**			
21.750	Butyrolactone	Faint, sweet, buttery	86, 56	[40,42]
22.097	δ-Valerolactone	Herbal, woody ^b^	56, 100	[43]
27.792	Pantolactone	Cotton candy ^b^	71, 57	[43]
29.721	δ-Octalactone	Sweet, coconut, creamy ^b^	99, 71	[43]
30.254	Dehydromevalonic lactone		88, 112	
	**Nitrogen Compounds**			
25.746	Benzyl nitrile	Bitter, almonds, spicy ^b^	117, 60	[43]
	**Pyrazines, piperazines**			
9.422	Methylpyrazine	Nutty, chocolate, cocoa	94, 67	[36,37,47]
11.557	2,5-Dimethylpyrazine	Cocoa, sweet chocolate, roasted nuts	108, 81	[45,51,52,53,54]
12.200	2,3-Dimethylpyrazine	Caramel, sweet chocolate, cocoa	108, 67	[9,37,40,51,52,53,54]
13.413	2-Ethyl-6-methyl pyrazine	Cocoa, roasted, green	121, 122	[45]
13.685	2-Ethyl-5-methylpyrazine	Nutty, raw potato, herbal, roasted	121, 122	[37,47]
				
13.838	Trimethylpyrazine	Cocoa, roasted nuts, sweet	122, 81	[9,36,39,45]
15.005	3-Ethyl-2,5-dimethylpyrazine	Earthy	135, 136	[47]
16.442	3,5-Diethyl-2-methyl pyrazine	Cocoa, chocolate, sweet	149, 150	[45]
16.611	Tetramethylpyrazine	Roasted, green, coffee, cocoa	54, 136	[36,39,45,55,56]
18.915	8-Methyl-1,2,4-triazolo [4,3-b] pyridazine		52, 134	
20.341	2-(3-Methylbutyl)-3,5-dimethylpyrazine		122, 135	
21.209	(E)-5-Methyl-2-(1-propenyl) pyrazine		134, 133	
22.846	2-Acetyl-3,5-dimethylpyrazine	Roasted, hazelnuts	60, 53	[40]
	**Pyridines**			
22.774	N-Acetyl-4H-pyridine		80, 123	
24.735	1-Acetyl-1,2,3,4-tetrahydropyridine		82, 83	
	**Pyrrol**			
6.500	1-Methylpyrrole	Woody ^b^	81, 80	[43]
24.060	2-Acetylpyrrole	Cocoa, hazelnut, Bread, licorice	94, 109	[36,45]
27.241	1-Methylpyrrole-2-carboxaldehyde	Roasted nutty ^b^	109, 108	[43]
29.640	1-Methyl-1,5-dihydro-2H-pyrrol-2-one	Ammoniacal ^b^	97, 60	[43]
	**Sulfur Compounds**			
12.424	Dimethyl trisulfide	Sulfurous	126, 79	[45]
26.072	Dimethyl sulfone	Sulfurous ^b^	79, 94	[43]
	**Terpenes, terpenoids**			
7.579	α-Pinene	Woody, herbal	93, 69	[57]
17.071	Linalool	Flowery, floral, fruity, lavender	71, 93	[9,15,16,37,40,42,52]
22.516	Epoxylinalool	Sweet, honey	68, 94	[45]

* Based on the HS-GC-MS analysis of chocolate bars: (i) bitter chocolate (70% cocoa), (ii) nibs chocolate (70% cocoa), (iii) milk chocolate (50% cocoa).^a^ The compound was earlier reported as a volatile compound in dark chocolate in the cited reference; ^b^ Flavor was reported in The Good Scents Company Information System website, a non-scientific publication [43].

**Table 2:** Key-VOCs of northern Peruvian chocolate.

Retention Time (min)	Fragments (*m*/*z*)	Molecular Formula	Volatile Organic Compound	Odor Description
16.611	54, 136	C_8_H_12_N_2_	Tetramethylpyrazine ^a^	Roasted, green, coffee, cocoa
17.346	57, 75	C_7_H_14_O_3_	Isobutyl lactate	Buttery, caramelly
18.915	52, 134	C_6_H_6_N_4_	8-Methyl-1,2,4-triazolo[4,3-b] pyridazine	*No flavor information*
19.309	55, 88	C_8_H_16_O_3_	2-Butoxyethyl acetate	Sweet, fruity
20.591	71, 116	C_6_H_12_O_2_	an isomer of Ethyl isobutyrate	Ethereal sweet, fruity
20.824	71, 116	C_6_H_12_O_2_	an isomer of Ethyl isobutyrate	Ethereal sweet, fruity
22.080	59, 71	C_4_H_8_O_3_	3-Hydroxybutanoic acid	*No flavor information*
22.516	68, 94	C_10_H_18_O_2_	Epoxylinalol	Sweet, honey
23.267	88, 89	C_8_H_14_O_4_	3,4-Dihydroxy-3,4-dimethyl-2,5-hexanedione ^a^	*No flavor information*
23.369	79, 107	C_8_H_10_O	α-Phenethyl alcohol	Sweet, floral
24.578	91, 104	C_10_H_12_O_2_	Phenethyl acetate ^a^	Sweet, floral

^a^ Compounds identity was confirmed by GC-MS/MS.

## Data Availability

Data is available upon request to Madina Mansurova, mmansurova@pucp.edu.pe; due to an agreement between the Pontificia Universidad Catolica del Peru and Teobroma Inver-siones S.A.C.

## Supplementary Materials

The following are available online at https://www.mdpi.com/2218-1989/11/2/71/s1, Figure S1: Relative intensities of each of the eleven volatile organic compounds present in the chocolates from northern and southern Peruvian regions, Figure S2: Comparative GC-MS chromatograms of two chocolate bar lots analyzed with three different SPME fibers: (i) divinylbenzene/polydimethylsiloxane (DVB/PDMS, red line), (ii) carboxen/polydimethylsiloxane (CAR/PDMS, blue line), and (iii) divinylbenzene/carboxen/polydimethylsiloxane (DVB/CAR/PDMS, black line).
