# Supplementary figures and images for: Mass Spectrometry-Based Flavor Monitoring of Peruvian Chocolate Fabrication Process
Source: Metabolites. 2021 Jan 26;11(2):71. doi: 10.3390/metabo11020071 (PMC7911988; doi:10.3390/metabo11020071)

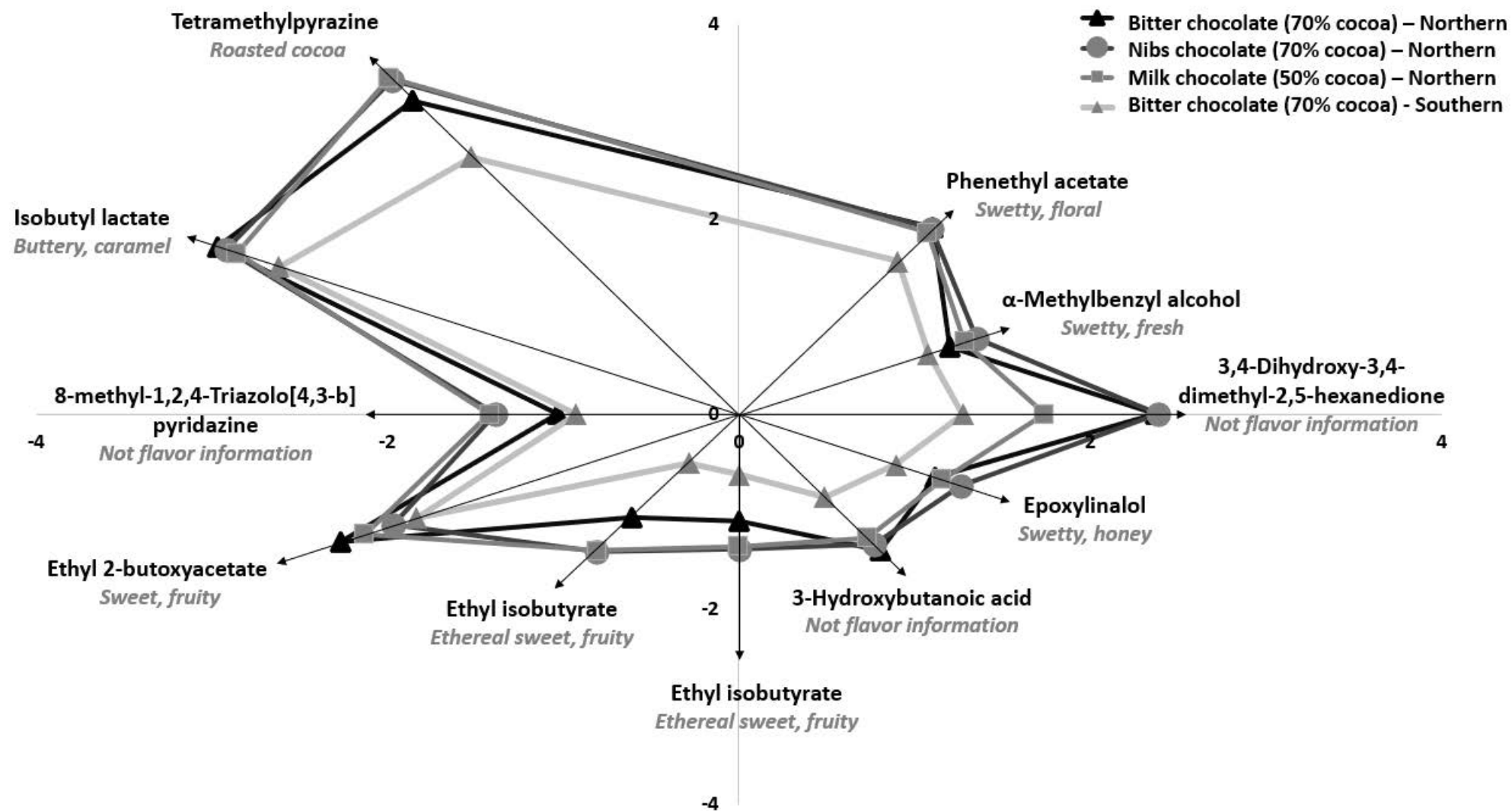

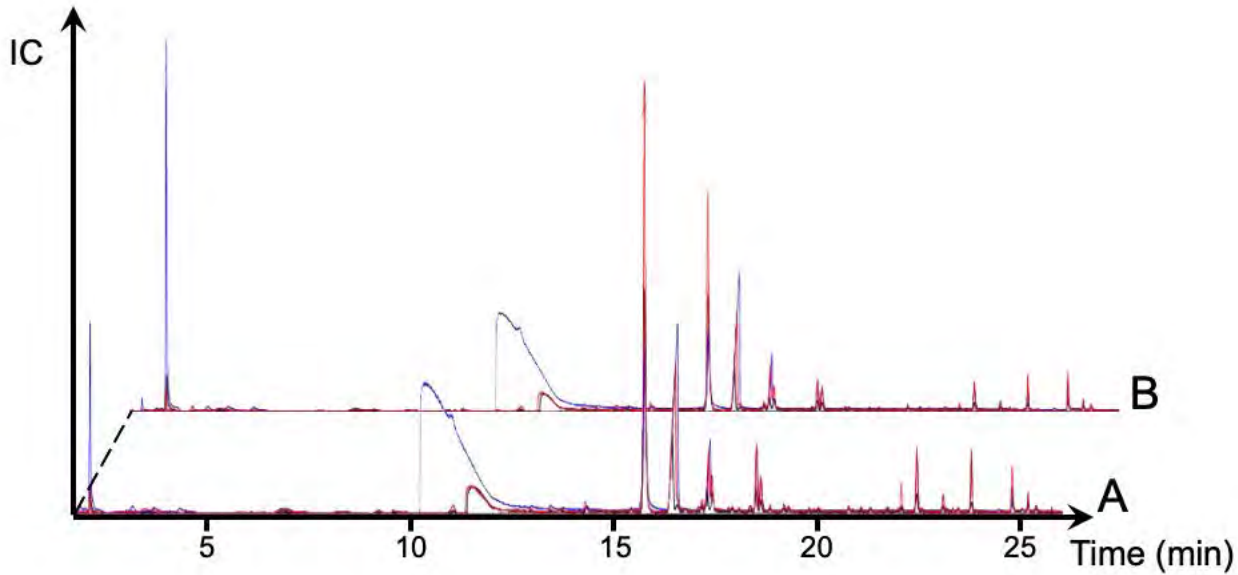

Supplement: Supplementary file 1 [file metabolites-11-00071-s001.pdf]
